# Low Family Support and Risk of Obesity among Black Youth: Role of Gender and Ethnicity

**DOI:** 10.3390/children4050036

**Published:** 2017-05-12

**Authors:** Shervin Assari, Cleopatra Howard Caldwell

**Affiliations:** 1 Department of Psychiatry, School of Medicine, University of Michigan, Ann Arbor, MI 48109, USA; 2 Center for Research on Ethnicity, Culture and Health, School of Public Health, University of Michigan, Ann Arbor, MI 48109-2029, USA; cleoc@umich.edu; 3 Department of Health Behavior and Health Education, School of Public Health, University of Michigan, Ann Arbor, MI 48109-2029, USA

**Keywords:** African Americans, youth, gender, ethnicity, parenting, family support, social support

## Abstract

Most studies on the role of family environment in developing risk of obesity among youth have focused on parenting behaviors that are directly involved in energy balance in regional, non-representative White samples. Using a national sample of ethnically diverse Black youth, the current study tested the association between low family support and risk of obesity. We also tested the heterogeneity of this association based on gender, ethnicity, and their intersection. We used data from the National Survey of American Life-Adolescent Supplement (NSAL-A), a national survey of Black adolescents in the United States. The study enrolled 1170 African American and Caribbean Black 13–17 year old youth. Obesity was defined based on the cutoff points of body mass index (BMI) appropriate for age and gender of youth. Family support was measured using a five-item measure that captured emotional and tangible social support. Age, gender, and ethnicity were also measured. Logistic regressions were utilized in the pooled sample, and also based on gender, ethnicity, and their intersection, to test the link between low family support and risk for obesity. Results: In the pooled sample, low family support was not associated with an increased risk of obesity (OR = 1.35, 95% Confidence Interval (CI) = 0.96–1.89). The association between low family support and risk of obesity was, however, significant among African American females (OR = 1.60, 95% CI = 1.01–2.55). There was no association for African American males (OR = 1.26, 95% CI = 0.82–1.92), Caribbean Black males (OR = 0.68, 95% CI = 0.01–54.85), and Caribbean Black females (OR = 0.78, 95% CI = 0.42–1.44). In conclusion, policies and programs that enable African American families to provide additional family support may prevent obesity among African American female youth. Future research should test the efficacy of promoting family support as a tool for preventing obesity among African American female youth.

## 1. Background

Family influences on risk of obesity among offspring are well established [1,2,3,4,5,6]. The majority of this evidence, however, is based on studies with local, non-representative, and mostly White samples [3,4,7]. Available research has mostly focused on family processes that are directly involved in energy balance, such as food consumption, physical activity, and sedentary life style issues [3,4,5,6,7,8,9,10,11,12,13]. As a result, less is known about the role of more general family processes such as family support, particularly among minority youth [2].

While family can be a part of the obesogenic environment for youth [14], it is unknown whether attributes that reflect overall family functioning (such as low family support)—but are not directly related to energy balance—also increase risk of obesity of the offspring. It is also still unknown whether such effects exist for Black youth [15,16]. Further, less information is available regarding whether the salience of low family support as a risk factor for obesity is universal across gender and ethnic groups [2]. Given the limits of existing knowledge, there is a need to develop a rich understanding of how ethnicity, gender, and their intersection alter the role of family environment on risk of obesity in Black youth [2].

Research has shown that race, ethnicity, gender, and their intersections alter psychosocial determinants of obesity [17,18,19,20,21,22,23,24,25,26,27,28,29,30,31,32,33]. Ethnic groups, for example, differ in the salience of psychosocial factors on obesity [29]. Gender also alters the relevance of family support for obesity [2]. The intersection of ethnicity and gender also alters psychosocial correlates of obesity [17,23,24]. In a recent study by Assari et al., 227 African American youth (109 male and 118 female) were followed for 12 years from age 20 to age 32. The study showed that among females, but not males, low maternal support during adolescence was a risk factor for an increase in body mass index (BMI) a decade later when they were young adults [2].

Researchers have found that the family environment affects behaviors that influence feeding, exercise, nutrition, and sedentary life style [15,34,35,36,37,38,39,40,41]; yet the existing knowledge regarding the obesity risk attributable to low sense of family closeness, support, and warmth is limited [35,36,37,38]. Although abuse, neglect, and harsh parenting (which operate as stressors) may increase risk of obesity [39,40], less is known about potential effects of poor family support on risk of obesity. While lack of emotional support may increase risk of obesity particularly for youth who live in a disadvantaged environment and deal with stress on a daily basis, the mechanism of the effects of such general family attributes is not directly through altering energy balance [2,41].

Using a national sample, the current study investigated the association between low family support and risk of obesity among ethnically diverse Black youth. We also tested possible heterogeneity of this association based on ethnicity, gender, and their intersection in an effort to understand more nuances about subgroup differences in Black youth. We hypothesized that low family support would be associated with higher odds of obesity in the pooled sample of Black youth. We also hypothesized ethnic by gender variation in this association, with a more salient role of low family support as a risk factor for obesity for African American females. Although we are not aware of any previous studies on ethnic by gender variation of this association among Black youth, low family support is shown to influence the risk factor for obesity in African American females [2].

## 2. Materials and Methods

### 2.1. Design and Setting

This was a cross-sectional study using data from the National Survey of American Life-Adolescent Supplement (NSAL-A) 2003 [42,43,44,45]. Funded by the National Institute of Mental Health (NIMH) and included in the Collaborative Psychiatric Epidemiology Surveys (CPES), the NSAL-A is a part of the National Survey of American Life (NSAL). The NSAL-A is one of the largest and most updated mental health surveys of Black youth ever conducted in the United States [44,45].

The NSAL-A received approval from the Institutional Review Board (IRB), University of Michigan, Ann Arbor. Informed consent was obtained from youth’s legal guardians. Assent was obtained from youth themselves. All respondents received monetary compensation ($50) for their participation.

### 2.2. Participants

#### 2.2.1. NSAL Sample

The NSAL, which consists of a nationwide survey of the African American, Caribbean Black, and non-Hispanic White adult population, is based on a stratified, multi-stage area probability sample of the non-institutionalized civilian population in the 48 contiguous states [46,47,48].

#### 2.2.2. Recruitment of Adolescents

The adolescent sample was drawn only from African American and Caribbean Black households. To recruit the adolescent sample, all African American and Caribbean Black households that included a participant in the NSAL-Adult survey were screened for eligible adolescents living in the household. Adolescents were then randomly selected from the provided list. When more than one eligible youth was living in the household, two adolescents were selected based on the gender of the 1st selected adolescent.

#### 2.2.3. NSAL-A Sample and Weight

As a result of the above procedure, the NSAL-A sample was non-independent. In response, the NSAL-A data were weighted to adjust for non-independence in selection probabilities within the households, as well as clustering, strata, and non-response rates across households and individuals. Finally, the NSAL-A weighted data was post-stratified to represent the national estimates based on gender and age among Black youth [44,45].

Additional details regarding NSAL sampling have been published elsewhere [46,47,48]. While African Americans were sampled from large cities or other urban and rural areas, Caribbean Blacks were exclusively sampled from large cities.

#### 2.2.4. Analytical Sample in the Current Study

Participants in the NSAL included 1170 ethnically diverse Black adolescents. The participants were African American (*n* = 810) or Caribbean Black (*n* = 360) youth who participated in the NSAL. The original adolescent sample consisted of 1193 cases, but 23 were dropped for analyses because they were 18 or older at the time of the interview. Thus, the resulting sample is 1170 African American (*n* = 810) and Caribbean Black (*n* = 360) youth ranging in age from 13 to 17 who were attached to the adult households (i.e., their parents participated in the NSAL study).

### 2.3. Interview

Approximately 82% of the data were collected via face-to-face home interviews. The remaining 18% of the data were conducted either entirely or partially by telephone interviews. Face-to-face interviews were computer-assisted personal interviews (CAPIs), a preferred interviewing technique when the survey instrument is long and complex. In CAPIs, interviewers use computers to input the respondents’ answers to questions. Interviews lasted 100 min on average and all interviews were performed in the English language. The overall response rate was 80.6%, with 80.4% for African Americans and 83.5% for Caribbean Blacks.

### 2.4. Measures

The study also collected demographic factors (i.e., age, gender, and ethnicity) as control variables. Obesity was measured using self-reports of height and weight and family support was also self-reported. All self-reported data were provided by youth themselves.

#### 2.4.1. Ethnicity

Youth ethnicity was identified according to the ethnicity of the parents who were living in the same household at the time of the survey. Parents self-identified as African American if they were Black and did not have ancestral ties to the Caribbean. Parents identified as Caribbean Black if they were Black and from a country included on a list of Caribbean countries presented by the interviewers, or if their parents or grandparents were born in a Caribbean country. The thirteen Caribbean countries included were: (1) Cuba, (2) the Dominican Republic, (3) Haiti, (4) the Bahamas, (5) Jamaica, (6) Trinidad and Tobago, (7) Dominica, (8) Saint Lucia, (9) Antigua and Barbuda, (10) Barbados, (11) Saint Vincent and the Grenadines, (12) Grenada, and (13) Saint Kitts and Nevis.

#### 2.4.2. Obesity

NSAL-A collected data on Body Mass Index (BMI) based on adolescents’ self-reported weights and heights. Although people tend to underestimate their weight and overestimate their height [49] which results in an underestimation of BMI [50], BMI based on self-reported weight and height has been shown to strongly correlate with BMI based on direct measures [51]. We used the standard definition of obesity in youth, which is based on the comparison of BMI with the 95th percentile BMI of the age- and gender- specific norms. We categorized our participants to obese (those with BMI % equal or greater than 95th percentile) versus non-obese (those with BMI % less than 95) individuals. Thus, obesity was treated as a dichotomous variable in this study [52,53].

#### 2.4.3. Family Support

The NSAL-A measured the amount of emotional and tangible support each adolescent received from the family using a 6-item survey. Items included How often do your family members (1) make you feel loved and cared for? (2) listen to you talk about your private problems and concerns? (3) express interest and concern in your wellbeing? (4) provide you with transportation? (5) help you financially? and (6) help you with child care or babysitting? If the answer was “Never”, participants were asked “Was that because you never needed help?” Response categories were: Very Often (score 1), Fairly Often (score 2), Not Too Often (score 3), and Never (score 4). We coded as missing if the participant had not needed support. We calculated a total score ranging from 5 to 20, with higher scores indicating low family support [54,55]. (Cronbach’s α = 0.750).

### 2.5. Statistical Analysis

To account for the complex survey design of the NSAL-A, we weighted the data using Stata 13.0 (Stata Corp., College Station, TX, USA). We applied Taylor series approximation technique for estimation of complex design-based standard errors (SE). Percentages reported in this study are weighted to depict nationally representative figures. As in the NSAL-A, the Caribbean Black sample is more clustered than the African American sample, thus SEs are systematically larger for Caribbean Blacks than for African Americans. This results in more conservative findings for Caribbean Blacks.

In this study, we used survey logistic regression for multivariable analysis by considering obesity as the outcome, family support (high score indicating low support) as the main predictor, age as the control, and ethnicity and gender as moderators. We ran a number of models to determine the moderating role of gender, ethnicity, and their intersection on our outcome of interest. In the first step, we ran the model with the pooled sample, controlling for the main effects of ethnicity and gender. In the next step, we conducted the analysis by ethnicity, gender, or their intersection subsamples. Adjusted odds ratio (OR), SE, and 95% confidence intervals were reported. *p*-Values less than 0.05 were considered statistically significant. We did not need to adjust *p*-value as we did not run multiple tests, but the same test in the pooled sample and subgroups.

## 3. Results

Participants were on average 15 years old (SD = 1.42) and had the following age distribution: 13–14 (*n* = 477, 40%), 15–16 (*n* = 441, 41%) and 17 (*n* = 252, 19%) years old. There were slightly more girls (*n* = 605, 52%) than boys (*n* = 563, 48%). Of the 1170 youth, 96% identified as a high school student. Family income ranged from US$ 0–520 thousand, with a median of US$ 28,000. Caribbean Blacks had a higher median income (US$ 32,250) compared to African Americans (US$ 26,000) (*p* < 0.001).

Table 1 describes the sample distributions for gender, ethnicity, age, family support, and obesity in the pooled sample, and based on ethnicity and gender.

Table 2 summarizes five logistic regressions. *Model 1* for the pooled sample showed that low family support was not associated with higher risk of obesity (OR = 1.35, 95% CI = 0.96–1.89). Subsequently; *Model 2* for African Americans; *Model 3* for Caribbean Blacks; *Model 4* for males; and *Model 5* for females also showed no significant associations between low family support and risk of obesity.

Table 3 summarizes four additional models for ethnicity by gender groups. The association between low family support and risk of obesity was significant among African American females (OR = 1.60, 95% CI = 1.01–2.55). We did not find such an association for African American males (OR = 1.26, 95% CI = 0.82–1.92), Caribbean Black males (OR = 0.68, 95% CI = 0.01–54.85), and Caribbean Black females (OR = 0.78, 95% CI = 0.42–1.44).

## 4. Discussion

In a national sample of Black youth, this study documented the role of low family support on risk of obesity. This study also showed heterogeneity of this association based on the intersection of ethnicity and gender. Although low family support was a risk factor of obesity for African American females (OR = 1.60), the association did not reach significance for young African American males, Caribbean Black males, or Caribbean Black females. For Caribbean Black males (OR = 0.68) and Caribbean Black females (OR = 0.78), although not significant, the direction of the association was reversed.

Our findings suggest that low family support may be a risk factor for obesity among African American females. Similar findings have been reported previously [2,19]. Jackson has attributed disproportionately high risk of obesity among African American women to the specific coping mechanisms that they use in dealing with stress [56]. African American women have a high tendency to turn to high calorie food in response to stress [57,58]. Future research is needed to understand how coping mechanisms mediate gender specific effects of stress on obesity among African Americans.

Only a handful of studies have tested the effect of general family processes on risk of obesity of offspring [2,35,36,37,38]. Only a small subset of these studies has enrolled Blacks or African Americans [2]. Based on our findings, in addition to family processes that are directly involved in eating, exercise, and life style, general family processes also influence risk of obesity. Youth who do not experience a positive family environment may be at an increased risk of obesity, as they may not benefit from the buffering effect of family support [59], particularly when they face stress and adversity on a daily basis.

In a recent longitudinal study of African American youth, low maternal support at baseline predicted an increase in BMI two decades later in females, but not males. The association remained significant net of socioeconomic status, family structure, and mental health (anxiety and depression). The study did not find any association between baseline paternal support and future obesity among African American males [2]. These findings fully support our results.

Based on our findings, the effect of low family support as a risk factor for obesity among Black youth varies based on the intersection of ethnicity and gender. Previous research has also shown that race, ethnicity, gender, and their intersections alter psychological correlates of obesity [22,26,29,30]. Differences in psychosocial determinants of obesity between African American males, African American females, Caribbean Black males, and Caribbean Black females have been previously reported [17,20,28].

These findings are in support of the intersectionality approach [60,61,62]. Intersections of race, ethnicity, culture, and social class shape family processes such as parenting styles and behaviors [63,64,65,66,67]. Emotional attachment and supportive relations between family members are also influenced by values, cultures, life conditions, and environment. The effects of these family processes are also shaped by the intersections of race, ethnicity, culture, gender, and social class [68,69,70,71,72,73,74,75].

A stronger effect of low family support on obesity risk of African American females is in line with the literature that shows that the gender of the offspring is a vulnerability factor to the effects of socioenvironmental and family characteristics [18,74]. Overall, parents are generally more affectionate with girls than boys [75,76]. Girls receive more emotional support from their parents compared to boys [77]. African American families are not an exception to this rule [67]. Boys and girls are also differently under influence of parenting and family environment [78,79,80,81,82]. This may explain why poor family relations may have a larger effect on obesity for African American females than males [83,84]. More studies have focused on the effect of family function on obesity of adults [16,85] or children [86,87] rather than youth [2]. In a study that used data from the Danish Twin Registry, comparison of same-sexed twin pairs discordant for BMI showed that perceived parental antipathy and neglect predict obesity [16]. Parenting support received from mothers and fathers also have differential effects on the mental health of male and female African American youth [18,74,88].

Our findings have implications for public policy and public health practice. Interventions such as Fathers and Sons [89,90] and Strong African American Families [91,92] are culturally sensitive, accepted by the community, and effective. Public policies should enhance opportunities to support positive family functioning specifically in the lives of African American women, which may reduce their obesity rates.

Our findings are relevant as obesity is an epidemic among African American women. In 2011, compared to Non-Hispanic Whites, African American women were 80% more likely to be obese, with four out of each five African American women being either overweight or obese [93]. Obesity increases the risk of a wide range of cardiovascular outcomes [94,95], while years lived with obesity increases the risk of death from cardiovascular causes, independent of BMI [95]. Obesity is also an established risk factor for hypertension [96], diabetes [97,98], cardiovascular disease [99], and stroke [100].

Although this study focused on low family support as a risk factor, our findings do not blame the victim. Family is a core value in Black culture [101,102]. Financial, economic, historical, and societal and political reasons have shifted their family structures from a more traditional nuclear form to an adaptable extended type. Adaptive family roles have been also taken by the family members. In addition to family, African American and Black families endorse a high sense of resilience. Along with other resilience factors such as racial identity, religiosity, and social networks, family support should be incorporated into interventions to improve health disparities [103,104,105]. Caldwell et al., provide a framework to enhance family support of Black families [74].

Family support can be enhanced on a large scale. There are policies and programs that effectively enhance supportive relations and the engagement of parents in the family [74,89,90,106]. Thus, family function should be considered as a target of national and local interventions; such investments have the capacity to also prevent obesity among African American women. Other researchers have also previously suggested that enhancing family and parental affection may be an effective strategy to prevent obesity [36]. More investment should be made on evidence-based family programs that enable families to have supportive relations in majority Black communities [74,89,90]. Examples of policy solutions are those which reduce parental stress and provide incentives for family engagement in the life of African American families. Such policies that enhance the family environment of African Americans will have health promoting effects on their offspring. Economic, financial, and employment policies are all particularly important, as most African American families struggle economically and live in socially disadvantaged neighborhoods.

To conclude, low family support does not have a universal role as a risk factor for obesity in all sub-groups of Black youth, with a more salient role for African American females than for African American males, Caribbean Black males, and Caribbean Black females.

## Figures and Tables

**Table 1:** Descriptive statistics in the pooled sample and based on ethnicity and gender.

	Gender						Ethnicity			
		All		Males		Females		African Americans		Caribbean Blacks
	% (SE)	95% CI	% (SE)	95% CI	% (SE)	95% CI	% (SE)	95% CI	% (SE)	95% CI
Gender										
Male	50.02 (0.02)	46.49–53.54					50.39 (0.02)	46.57–54.20	44.78 (0.02)	39.98–49.68
Female	49.98 (0.02)	46.46–53.51					49.61 (0.02)	45.80–53.43	55.22 (0.02)	50.32–60.02
Ethnicity										
African Americans	93.37 (0.01)	91.89–94.60	94.07 (0.01)	92.69–95.20	92.68 (0.01)	90.64–94.31				
Caribbean Blacks	6.63 (0.01)	5.40–8.11	5.93 (0.01)	4.80–7.31	7.32 (0.01)	5.69–9.36				
Obesity										
Non–Obese	75.31 (0.02)	71.13–79.06	75.92 (0.03)	70.51–80.62	74.69 (0.02)	69.39–79.34	75.08 (0.02)	70.62–79.06	78.54 (0.06)	63.68–88.42
Obese	24.69 (0.02)	20.94–28.87	24.08 (0.03)	19.38–29.49	25.31 (0.02)	20.66–30.61	24.92 (0.02)	20.94–29.38	21.46 (0.06)	11.58–36.32
	m (SE)	95% CI	m (SE)	95% CI	m (SE)	95% CI	m (SE)	95% CI	m (SE)	95% CI
Age	14.98 (0.06)	14.86–15.10	14.98 (0.07)	14.84–15.12	14.98 (0.09)	14.80–15.15	14.96 (0.07)	14.83–15.10	15.22 (0.06)	15.09–15.35
Family Support (Low)	3.55 (0.01)	3.52–3.58	3.55 (0.02)	3.51–3.59	3.55 (0.02)	3.51–3.59	3.55 (0.02)	3.52–3.58	3.55 (0.03)	3.49–3.61

Data: National Survey of American Life-Adolescent Supplement (NSAL-A); Sample size: 1170 African American and Caribbean Black 13–17-year old youth. SE: Standard Error; CI: Confidence Interval, m: mean.

**Table 2:** Logistic regression in the pooled sample and based on race and gender.

	OR (SE)	95% CI	*p*
All			
Ethnicity (Caribbean Blacks)	0.84 (0.30)	0.41–1.72	0.628
Gender (Female)	1.07 (0.17)	0.77–1.48	0.682
Age	0.92 (0.06)	0.81–1.04	0.162
Family Support (Low)	1.35 (0.22)	0.96–1.89	0.082
Intercept	0.38 (0.45)	0.04–4.09	0.418
African Americans			
Gender (Female)	1.09 (0.19)	0.77–1.54	0.614
Age	0.91 (0.06)	0.80–1.04	0.173
Family Support (Low)	1.39 (0.23)	0.98–1.96	0.061
Intercept	0.35 (0.43)	0.03–4.34	0.398
Caribbean Blacks			
Gender (Female)	0.75 (0.51)	0.18–3.19	0.675
Age	1.00 (0.09)	0.82–1.22	0.990
Family Support (Low)	0.77 (0.68)	0.11–5.15	0.768
Intercept	1.12 (4.20)	0.00–3554.5	0.977
Males			
Ethnicity (Caribbean Blacks)	0.99 (0.16)	0.72–1.37	0.955
Age	0.85 (0.06)	0.74–0.99	0.035
Family Support (Low)	1.23 (0.27)	0.79–1.90	0.346
Intercept	1.59 (2.20)	0.10–26.15	0.741
Females			
Ethnicity (Caribbean Blacks)	0.69 (0.44)	0.19–2.51	0.562
Age	0.99 (0.10)	0.81–1.20	0.902
Family Support (Low)	1.53 (0.33)	0.99–2.36	0.054
Intercept	0.09 (0.15)	0.00–2.44	0.148

Outcome: Obesity; Data: National Survey of American Life-Adolescent Supplement (NSAL-A); Sample size: 1170 African American and Caribbean Black 13–17 year old youth. OR: Odds Ratio.

**Table 3:** Logistic regression in the pooled sample and based on the intersection of race and gender.

	OR (SE)	95% CI	*p*
African American Male			
Age	0.84 (0.06)	0.72–0.98	0.032
Family Support (Low)	1.26 (0.26)	0.82–1.92	0.275
Intercept	1.75 (2.46)	0.10–31.16	0.692
Caribbean Black Male			
Age	1.15 (0.13)	0.90–1.46	0.251
Family Support (Low)	0.68 (1.40)	0.01–54.85	0.855
Intercept	0.16 (1.38)	0.00–1.59	0.835
African American Female			
Age	0.99 (0.10)	0.81–1.22	0.940
Family Support (Low)	1.60 (0.37)	1.01–2.55	0.050
Intercept	0.07 (0.12)	0.00–2.37	0.135
Caribbean Black Female			
Age	0.90 (0.08)	0.74–1.10	0.294
Family Support (Low)	0.78 (0.22)	0.42–1.44	0.402
Intercept	2.76 (5.77)	0.03–243.44	0.634

Outcome: Obesity; Data: National Survey of American Life-Adolescent Supplement (NSAL-A); Sample size: 1170 African American and Caribbean Black 13–17 year old youth.
